# Operatic Singing Biomechanics: Skeletal Tracking Sensor Integration for Pedagogical Innovation

**DOI:** 10.3390/s25154713

**Published:** 2025-07-30

**Authors:** Evangelos Angelakis, Konstantinos Bakogiannis, Anastasia Georgaki, Areti Andreopoulou

**Affiliations:** Laboratory of Music Acoustics and Technology (LabMAT), Department of Music Studies, National and Kapodistrian University of Athens, Panepistimioupoli, Ilisia, 15784 Athens, Greece; k.bakogiannis@music.uoa.gr (K.B.); georgaki@music.uoa.gr (A.G.)

**Keywords:** skeletal tracking, operatic singing, vocal pedagogy research, voice biomechanics, multisensor recording

## Abstract

Operatic singing, traditionally taught through empirical and subjective methods, demands innovative approaches to enhance its pedagogical effectiveness today. This paper introduces a novel integration of advanced skeletal tracking technology into a prototype framework for operatic singing pedagogy research. Using the Microsoft Kinect Azure DK sensor, this prototype extracts detailed data on spinal, cervical, and shoulder alignment and movement data, with the aim of quantifying biomechanical movements during vocal performance. Preliminary results confirmed high face validity and biomechanical relevance. The incorporation of skeletal-tracking technology into vocal pedagogy research could help clarify certain technical aspects of singing and enhance sensorimotor feedback for the training of operatic singers.

## 1. Introduction

Operatic singing demands a voice with accurate control and efficiency but also a voice that can resonate with the audience, a feat that demands many years of rigorous training. Over its course through the centuries, vocal pedagogy has evolved, shaped by one main goal: to refine and enhance vocal technique, constantly balancing artistry with precision. Traditional methods, while foundational, often seem to be lacking the precision and adaptability required to meet the fast-paced socio-technological shifts and the demands of contemporary operatic performance [1]. Therefore, as technology continues to evolve, so too must the methods for teaching the art of operatic singing, necessitating innovative tools that bridge the gap between traditional empirical pedagogy and modern scientific understanding. Yet, despite the centuries of accumulated knowledge, critical questions regarding the singing voice remain points of speculation or disagreement between teachers and unanswered by science alike [2]. Many basic aspects of singing technique and education are still matters of subjective opinion. During the last few decades, vocal pedagogy has somewhat shifted toward incorporating science-based practices, acknowledging the biomechanics and cognition of the human voice [3].

Part of the biomechanics relevant to operatic singing includes posture, specifically cervical, shoulder, and spinal alignment, which can, among other factors, significantly affect breathing, laryngeal position, and muscular tension around the larynx. Recognizing these biomechanical interactions is crucial for both understanding vocal mechanics and enhancing pedagogical methods.

The innovative features of the present work lie in the fact that, to the best of our knowledge, it is the first operatic voice research attempt to integrate the latest skeletal-tracking camera technology as a tool for capturing and analyzing posture and movement relevant to operatic singing research. This innovation uniquely enables precise tracking of posture and related movements, providing detailed data on spinal/postural, cervical, and shoulder alignment and movements. Moreover, it allows for the storage, processing, and synchronization of these biomechanical data streams alongside spectral, glottal, laryngeal, and breathing data.

The importance of integrating such skeletal tracking sensors into operatic pedagogy is ever more important, considering contemporary lifestyle and kinetic habits. A lifestyle with elevated stress levels often contributes to increased muscular tension, resulting in altered breathing patterns, movement tendencies, and postural deviations. Additionally, everyday body posture and neck alignment practices while driving or interacting with computer and mobile device screens further add to these issues.

These contemporary lifestyle changes showcase the necessity for evidence-based research in singing pedagogy. A systematic review study conducted across 15 databases reported that “Research in classical singing training has not yet developed an evidence-based framework for classical singing training,” despite such frameworks existing in analogous fields like education and sports [4]. Indeed, research in exercise and sport sciences has effectively established evidence-based frameworks that provide structure to teaching and learning processes, outline strategies conducive to performance improvement, and assist learners in achieving motor skill acquisition and functional independence [4]. Herbst, in [2], addresses this very gap, questioning whether vocal pedagogical methods specifically target known vocal subsystems, muscle groups, and physical/physiological functions, or if these practices are merely traditional habits employed because they appear effective. Addressing this question should be a central driving notion for singing voice scientists and pedagogues alike [2].

Historically, vocal pedagogy has consistently acknowledged the importance of biomechanical and physiological aspects. Manuel García I, author of “Exercises Pour La Voix” (1819–1822), highlighted practical methods consisting of 340 vocal exercises, emphasizing—among others—the importance of body posture [5]. Other influential pedagogues, such as Francesco Lamperti (1813–1892) and his son Giovanni Battista Lamperti (1839–1910), similarly advocated the “noble posture” of the seventeenth and eighteenth centuries singers [6].

This study explores the adoption of the Microsoft Kinect Azure DK, Microsoft Corporation, Redmond, WA, USA for tracking operatic singing biomechanics and validating its relevance for pedagogical research. The primary aims are to verify its biomechanical relevance and establish its validity in capturing accurate posture, cervical, and shoulder movement data during vocal performance. By doing so, this research aims to provide a way to record pedagogically valuable operatic singing biomechanics in a quantifiable manner, contributing to a more objective understanding of singing technique.

## 2. State of the Art

Vocal pedagogy history features numerous periods and figures where it meets and fuses with science and technology. However, our era seems to be the first one to herald a conscious, wider outreach of vocal teachers and students seeking answers in the scientific research domain and beyond. A growing number of pedagogues and institutions shift a significant part of their focus toward science and/or technology. This latter trend is usually referred to as “Science-informed Vocal Pedagogy” [7], or “Fact-based Vocal Pedagogy” [8,9], but also with many other similarly descriptive-of-the-manner titles. During the last few years, there has been an ever-growing spread of the term “Evidence-Based Vocal Pedagogy” [10], often abbreviated as ‘EBVP,’ a teaching model that also includes the utilization of scientific evidence relevant to voice production.

Recent research in vocal pedagogy has begun to leverage advanced motion-tracking technologies to objectively analyze singers’ posture and movements. For example, Luck and Toiviainen used a high-resolution motion capture system to extract kinematic features of classical singers’ bodies, revealing how head and torso alignment variations can measurably affect vocal output [11]. In a recent study, Moreno utilized a gold-standard—though slightly more invasive—method, namely the Vicon optical tracking system, to quantify alignment at certain key anatomical points (such as the atlanto-occipital joint, shoulders, and lumbar spine) [12]. The work studied singers before and after somatic training and found significant postural improvements (particularly in head-neck and lumbar alignment) following targeted body-mapping instruction, thus demonstrating the value of precise skeletal data in assessing pedagogical interventions.

Outside the singing studio, fields such as sports science and dance have widely adopted both marker-based and markerless motion capture to enhance technique training. Bawa et al. identify the Microsoft Kinect as a viable, noninvasive tool for capturing kinematic features in gait and postural assessments, with applications ranging from physiotherapy to athletic performance analysis [13]. In music and performing arts, researchers have similarly explored the potential of using the Kinect system for tracking purposes; Gillies et al. developed a Kinect-based system to give real-time feedback on musicians’ playing posture [14], and dance technologists have used the Kinect skeletal tracking to recognize complex choreography patterns via posture data (e.g., folk dance sequences in [15,16]).

The latest generation Azure Kinect DK offers substantial improvements in accuracy and scope, tracking 32 body joints (including detailed head/neck markers) and yielding kinematic measurements comparable to gold-standard laboratory systems. Experimental assessment of the device proved “the feasibility of applying the Azure Kinect to the biomechanical monitoring of human motion in several fields” [17]. Empirical evaluations show that the Azure Kinect achieves high agreement with marker-based motion capture for upper-body joint motion and postural metrics [16], while providing greater flexibility for real-world use. Notably, a recent validation reported sub-centimeter limb-length accuracy versus dual-energy X-ray imaging [18], illustrating the precision of the sensor. Despite these advances, the integration of full-body biomechanical tracking in operatic singing pedagogy remains nascent. By drawing on proven tools such as Kinect-based skeletal tracking—as widely utilized in sports and dance—vocal pedagogy research can begin to quantitatively capture head, neck, shoulder, and spinal posture during singing.

In order to help address some of the problems reported in operatic singing pedagogy, as analyzed in a previous work [1], this paper proposes an addition to the arsenal of vocal research: the ability to record pertinent singer movements as quantifiable data. By capturing data from distinct parts of the vocal mechanism and synchronizing them with the vocal output, we aim to provide an initial framework for analyzing the manner in which some physiological variables influence vocal performance. Ultimately, research utilizing such data could pave a pathway to transforming the art of singing pedagogy from a mostly empirical craft into a more objective discipline.

## 3. Materials and Methods

### 3.1. Study Description

The skeletal tracking setup presented in this study was used to support the experimental part of a larger project led by the Haute École de Musique de Genève. The study attempted to explore the National School of operatic singing and vocal pedagogy system in Kazakhstan, in comparison to Central European systems, through historical, musicological, empirical/perceptual, and scientific data. The experiment was conducted in three distinct locations, namely the Kazakh National Conservatory in Almaty and the Haute école de musique de Genève (HEM) in Geneva and Neuchâtel. Students of singing of the above institutions were recruited for participation in the recordings. A total of 28 students of singing (henceforth referred to as singers) were recorded, most of them near-graduate, graduate, or post-graduate students, and some young professionals. Reported singer genders were: male = 16 (57.14%) and female = 12 (42.86%). Median age of the singers participating in the experiment was 22.5 (IQR: 20.5–28, min–max: 19–36), and the median years of vocal studies of the singers was 6 (IQR: 5–10, min–max: 2–21). Seventeen of the participants (60.71%) conducted their studies in Kazakhstan and eleven (39.29%) in Switzerland.

A latest-technology skeletal tracking camera was added to the list of available sensors of an evolving recording prototype for operatic voice research to innovatively allow for posture and pertinent movement tracking. The remaining sensors integrated into this prototype were: a condenser microphone, an electroglottograph (EGG), two distinct respiratory effort transducers, and HD video.

### 3.2. Advantages of Kinect Azure for Studio-Based Operatic Singing Research

Considering the purpose for use, the design of the prototype system at the making emphasized portability, venue adaptability, low invasiveness, low noise/interference, modularity, comprehensive coverage of the vocal mechanism, quality and precision, and future proofing. Following these criteria, the skeletal tracking sensor that was incorporated into this stage of the prototype was the new Microsoft Kinect version, Kinect Azure DK. This model integrates advanced AI sensors, including a depth camera and a seven-microphone circular array. It also features a machine learning model used for skeleton and joint tracking in a 3D space and an increased number of tracked joints in comparison to previous models.

The choice of the Kinect Azure DK for skeletal tracking in this operatic voice research prototype demonstrates clear advantages over wearable Inertial Measurement Unit (IMU)-based systems when evaluated against the specific goals of low invasiveness, cost-effectiveness, ease of use in a vocal studio setting, and pedagogical applicability. Unlike wearable IMUs—some of which may require multiple sensors to be strapped to the singer’s body, often with time-consuming calibration—Kinect offers a fully non-contact, camera-based solution that eliminates participant discomfort and preserves natural singing posture.

In the context of operatic singing research, where singers are asked to sing in their usual studio recording or concert-like conditions, the usual result is an upright pose facing a microphone, with slow movement of small extent and range, rather than abrupt, dynamic whole-body movements. The slow movement speed allows for this type of sensor to be used with a non-significant risk of failing to detect meaningful postural adjustments, given that the Kinect Azure reports data at a rate of 30 Hz. Even considering an effective half of this rate to control for the Nyquist effect, a sampling rate of 15 Hz seems unlikely to limit the validity of posture assessment in such scenarios.

In such controlled studio conditions with consistent lighting and clear line-of-sight, the depth camera and AI-driven joint tracking of Kinect provide sufficient precision to capture meaningful postural and head–neck alignment data relevant to vocal technique. It is also important that the Kinect has a single-device, tripod-mounted setup that can be rapidly deployed in different studios without the need for specialized training, enabling accurate data collection by non-expert operators, such as vocal teachers or the students themselves. Beyond that, its low cost relative to professional-grade wearable IMU systems dramatically improves research accessibility. Another advantage of using this device is the fact that it can be powered and transmit data through a single cable, thus (a) eliminating the need for an external power supply and (b) decreasing interference and noise in the signals. For example, in this particular experimental setup, the Kinect Azure was connected to the respective laptop through the USB-C port, using a Cable Matters [Intel Certified] 40 Gbps Active Thunderbolt 4 Cable, which was also used to adequately power the device. Overall, the Kinect Azure DK aligns optimally with the prototype’s design, making it an especially suitable choice for collecting high-quality research data in vocal studios and for supporting innovative pedagogical tools.

### 3.3. Kinect Max/MSP Patch Creation

**Figure 1:**
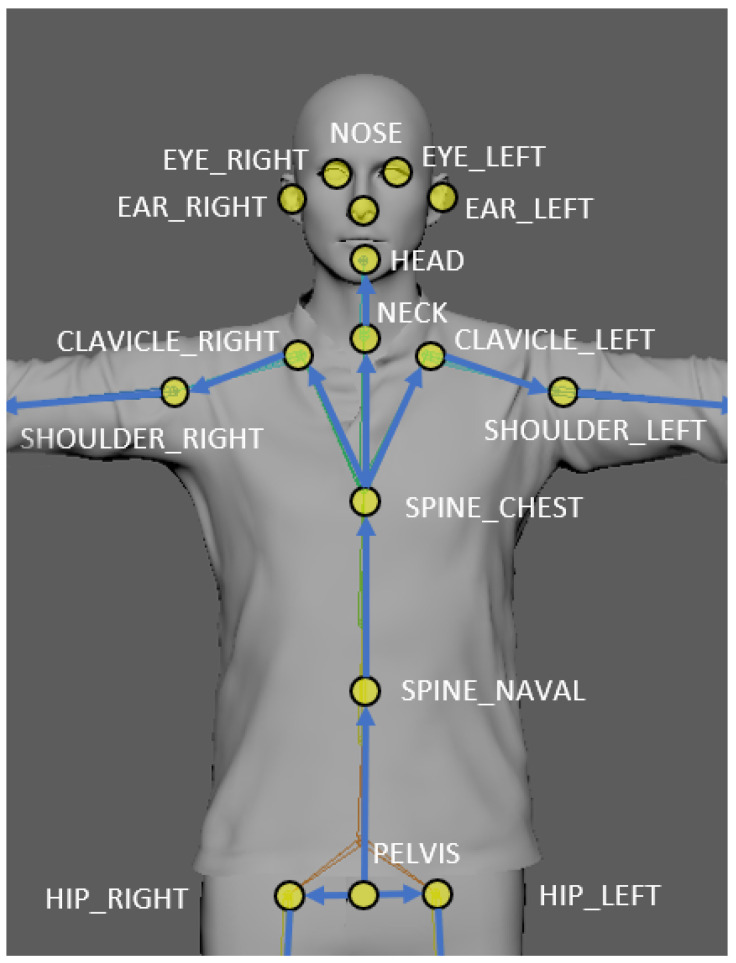
Kinect Azure DK skeleton tracking joints tracked and recorded in this work (head, right eye, left eye, right ear, left ear, nose, neck, spine chest, right shoulder, left shoulder, navel, pelvis, right hip, left hip). Part of the figure “Joint hierarchy” from Microsoft Kinect Documentation. ©Microsoft. Used under terms of use/fair use [20].

A commercial Max/MSP v8.0 object that could effectively read Azure Kinect DK output data [dp.kinect3] [19] was used to develop a Max/MSP patch that can read the 15 selected-as-pertinent Kinect body joints (see Figure 1) and store them in a text (.txt) file. Each line of the text file contains data regarding a single time frame. All text file lines start with two distinct timestamps (one from the Kinect and one from the computer internal clock), followed by the Kinect microphone sound level (recorded for offline synchronization purposes). These are followed by 15 sets of 8 digits (one set for each of the 15 body joints recorded by the patch). The above sets contain (1) the skeletal tracking coordinates (3 digits), (2) the skeletal tracking confidence value (possible values: 0.0, 0.5, and 1.0, corresponding to the confidence of the AI system on the output coordinate values of the joint), and (3) the skeletal tracking joint orientation values (4 digits).

The skeletal tracking joint orientation data can be in the form of quaternions (4 floats) or a 4 × 4 matrix (16 floats), and each of these can be output in hierarchical or absolute rotation. Given the number of 15 joints recorded, a 4 × 4 orientation matrix would require the storage of 15×16=240 extra floats per 1/60 s just for the orientation information. This would assume even more resources and computing power than the already extremely demanding Kinect Azure requirements for skeletal tracking. Instead, orientation was opted to be output and recorded as a 4-float quaternion set for each joint, using hierarchical rotation. This solution also ensured smooth transitions, free of gimbal lock.

Quaternions offer an alternative method for representing orientation or rotations in 3D space through a set of only four numbers. They are capable of precisely defining any three-dimensional rotation around an arbitrary axis, avoiding issues like the loss of one of the three degrees of freedom (pitch, yaw, and roll) in the representation of orientation (an issue referred to as gimbal lock). They are especially useful in cases similar to skeleton tracking, like fields involving spatial transformations. Unlike rotation matrices, which require nine numbers, quaternions efficiently provide all the information needed to rotate a vector using only four numbers.

However, the use of quaternions was deemed too much of an overstretch in the scope of a musicological/musico-technological research. The actual utilization of accurate joint orientation data would probably be required in cases of multiple distinct gesture-following. Instead, the movements pertinent to this project were analyzed using the Cartesian coordinates of the joints involved in each movement. Nevertheless, quaternion joint orientation values were recorded to enable future data meta-analysis, making a step towards a future-proof prototype.

To control for miscalculated data from the skeletal tracking, such as noise, a smoothing filtering method was applied. The five-parameter method used was based on a Holt double-exponential filter with the five Holt parameters of smoothing, correction, prediction, jitter-reduction radius, and max-deviation radius. A medium smoothing intensity was selected, applying values 0.5, 0.1, 0.5, 0.1, and 0.1 to the respective five Holt parameters, as suggested in the documentation. A smoothing value of 0.5 produces results with balanced smoothing versus latency. A correction of 0.1 smooths more towards the raw data values, while the selected prediction value predicts 0.5 frames of future position with an unlikely chance of overshoot, even in the case of a quick change in data (fast movement). Finally, a 0.1-meter jitter-reduction radius was selected, as well as a 0.1-meter max-deviation radius, which defines the maximum filtered position deviation from raw data.

Although the Kinect Azure DK has a maximum sampling rate of 30 frames per second, the [dp.kinect3] was requested to output values at a rate of 60 Hz. This resulted in duplicate data but helped mitigate data losses. Data collection at a rate of 60 frames per second results in text files with 60 lines of data for each second of skeletal-tracking recording time. At the press of the data acquisition/recording ‘stop’ button, the text lines are automatically saved in a file that bears as a filename the exact time and date of the recording commencement.

### 3.4. Data Collection

Prior to measurements, singers were contacted and given written instructions, including suitable attire, to ensure sensor precision regarding quality of data acquired and comfort. Upon arrival, singers read the description of the experimental process and pertinent information and were then asked to sign informed consents, as well as to complete a demographics and vocal health questionnaire.

A laptop was dedicated to reading in and recording the skeletal tracking data from the Kinect Azure DK camera. The measurement protocol for each singer started with a placement and calibration process of the Kinect camera. The camera was positioned such as to form a 45° angle between the skeleton tracking sensor’s line of sight and the line passing from both hip joints of the singer. This position was found to yield the best possible tracking results out of all viable available options, since a 90° angle would be occluded by the utilized microphone ‘booth,’ and a 0° angle (where the camera faces exactly side-on) the torso itself would occlude some joints on the far side of the body (such as the opposite shoulder, eye, and ear) [17]. Furthermore, a 0° angle camera placement would in fact not be feasible in a typical rectangular room with participants being recorded standing at 90 cm from the wall corner (opted for sound recording purposes), as the side-on placement would put the camera too close to the corner to maintain the necessary capturing distance from the singer. The custom Max/MSP patch was used to record the coordinates and orientation of the 15 pertinent body joints (head, right eye, left eye, right ear, left ear, nose, head center, neck, thorax, right shoulder, left shoulder, navel, pelvis, right hip, left hip), as seen in Figure 1. These data were automatically exported along with their corresponding timestamps and sound level values at a sample rate of 60 Hz. Video recordings of all experiment trials were made in 1080 p 30 fps using an iPhone 13 Pro and were later used as ground truth for validation purposes.

Each measurement started and was concluded with a short synchronization sequence, which consisted of three hand claps, followed by three small cough-like glottal attack sounds, produced simultaneously with an abdominal muscle inward activation, and a small, sharp downward head bend. This latter event was selected, as it provides information recorded by all sensors utilized and could thus be used for data synchronization verification. A large pause of about 10 s was introduced between the first and second clap, during which singers were asked to stand, in complete silence, in what they considered their personal optimal upright posture. Skeletal tracking data from this pause was used to determine each user’s reference posture.

The data collection phase commenced with data synchronization events, followed by the recording of (1) various vocal exercises, (2) the two initial phrases of the aria ‘Caro mio ben,’ (3) a romantic-era Italian aria of choice, and (4) a song of choice in the native tongue of each singer. The procedure concluded with the ending synchronization-event sequence. The whole process lasted between 17 and 29 min for each participant, depending on the duration of their selected musical pieces and calibration ease.

### 3.5. Data Post-Processing

Offline processing of the collected data was facilitated by a custom script written in MATLAB R2023a. The first step involved synchronizing the recorded data streams across the various sensors. This was achieved with the use of predetermined reference events that were inserted into the experimental protocol, such as claps and coughs, which are easily trackable and suitable as synchronization indices.

To ensure the quality and reliability of the skeletal tracking signals, the following post-processing steps were applied:

- Projection of joint coordinates onto task-relevant planes (e.g., XZ or XY) to reduce dimensional noise.
- Outlier detection and correction using a high threshold factor, ensuring that transient tracking errors are excluded.
- Mean-referencing to a stable interval and dynamic normalization, reducing the effect of inter-trial or inter-subject variability in baseline alignment.
- Moving average smoothing to reduce jitter in angle-based estimations, helping towards the elimination of minor fluctuations caused by the Kinect’s depth resolution noise.

More specifically, to address inconsistencies in body rotation between joints in the collected skeleton tracking data, a function was developed to adjust the data so that the skeleton consistently faces the camera perpendicularly. This was achieved by using the positions of the two hip joints to ensure they consistently form a 90° angle with the camera line-of-sight axis. The natural upright position of the singers’ bodies, which was documented individually during the calibration phase, when participants were asked to remain still, was also taken into consideration during this process.

To handle occasional tracking artifacts, an outlier detection step was implemented to identify values that deviate substantially from the typical range of the signal. Specifically, any data point that deviated more than eight times the typical spread around the median was considered an outlier and was flagged. This conservative limit ensures that only prominent spikes are corrected, while normal data variation is preserved. Detected outliers are then adaptively adjusted: values above the mean of the non-outlier points are slightly scaled down (mean × 1.2), while those below the mean are slightly increased (mean ÷ 1.2). This approach preserves the overall signal trend while minimizing the presence of unrealistic data spikes.

To ensure comparability across different singers and trials, each movement signal was mean-referenced to a stable baseline interval—defined by a neutral posture segment that the singer was asked to assume and sustain for a given time duration. The mean of this reference segment was subtracted from the entire signal to align it to a common zero baseline, removing static offsets. After mean-referencing, the signal was dynamically normalized to a unit range (−1 to 1) to account for differences in body size or individual motion extent, ensuring that movement patterns could be compared across participants on the same relative scale.

To further reduce small, rapid fluctuations in the calculated joint angles—often caused by minor variations in Kinect’s depth sensing—a simple 10-sample moving average filter was applied to each signal, effectively dampening jitter and ensuring that gradual, genuine posture changes are preserved while high-frequency noise is suppressed. This step improves the clarity of the extracted movement trends, making them more interpretable for pedagogical feedback and analysis.

### 3.6. Definition of Tracked Movement Features

MATLAB was also used to process the skeletal tracking data and output 7 distinct data streams of specific movement types. These movements were selected upon consultation with internationally acclaimed singing teachers as the most appropriate and relevant for study. They were clearly visible movements that could either impact vocal production or be good indicators of a technical, habitual, or physiological issue that could impede the optimal function of the kinetic mechanisms of the voice. These selected movements were body (spinal) posture, up-down head bend, left-right head turn, horizontal front-back head movement, right shoulder up-down, left shoulder up-down, and shoulder front-back (kyphosis-backward stretch).

1. Body posture: overall forward or backward spinal bending of the upper body relative to pelvis.
2. Up-down head bend: vertical flexion of the head relative to the torso axis.
3. Left-right head turn: rotational movement of the head to the left or right about the neck axis.
4. Horizontal front-back head movement: anterior-posterior displacement of the head relative to spinal alignment.
5. Shoulder up-down: vertical displacement of each shoulder’s height relative to the spine-pelvis axis.
6. Shoulder front-back (kyphosis-backward stretch): anterior rounding or posterior retraction of both shoulders relative to the spine.

To numerically describe these movements, the angles formed between distinct body lines were calculated (where each line is defined by two joints), and the relative changes in joint coordinates were assessed. Each line was treated as a vector derived from a pair of joints, so that any angular change between body segments could be described as the angle between two such vectors.

The angles formed between vectors were analyzed through their projection onto the azimuth and elevation planes. Prior to this analysis, the coordinates were transformed from the Cartesian to the spherical coordinate system. Each line was represented as a vector (denoted as $\vec{A}$ and $\vec{B}$), and the angle between vectors was calculated using the following formula:

$$\theta =\cos^{-1}\left(\frac{\vec{A}\cdot \vec{B}}{\|\vec{A}\|\cdot \|\vec{B}\|}\right)$$

where $\vec{A}\cdot \vec{B}$ denotes the dot product of the vectors, and $\|\vec{A}\|$, $\|\vec{B}\|$ their respective magnitudes (Euclidean norms). The direction of the angle (i.e., whether it represented a positive or negative rotation on the projected plane) was determined based on the sign of the cross product $\vec{A}\times \vec{B}$.

The joint pairs used to define the vectors $\vec{A}$ and $\vec{B}$ for each movement type are listed below:

1. Body posture: $\vec{A}$ = pelvis to spine, $\vec{B}$ = spine to center of the shoulder.
2. Up-down head bend: $\vec{A}$ = pelvis to spine, $\vec{B}$ = head to nose.
3. Left-right head turn: $\vec{A}$ = head to nose, $\vec{B}$ = right hip to left hip (as a body-centered reference line).
4. Horizontal front-back head movement: $\vec{A}$ = spine to head, $\vec{B}$ = head to nose.
5. Shoulder up-down: $\vec{A}$ = pelvis to spine, $\vec{B}$ = spine to shoulder (left or right, depending on side tracked).
6. Shoulder front-back (kyphosis or backward stretch): $\vec{A}$ = left shoulder to spine, $\vec{B}$ = spine to right shoulder.

### 3.7. Reliability of the Tracking System

While the skeletal tracking system was under development, its reliability was first validated through the capture of simple, isolated movements and then tested with more complex and faster motions. Before the main data collection, the first author—who is both a professional operatic singer and an experienced vocal coach—performed all target movements in an intentionally exaggerated manner as a pilot validation stage. This approach ensured that all the subtle, technique-related movements expected during natural studio or concert-like singing conditions would be reliably captured. The same test movements were also performed by the other authors, who vary in body size, to confirm robustness across different physiques.

By comparing the visual output from synchronized video recordings with the graphical results generated by the processing script (based on the Kinect data), the team verified that the extracted movement features—such as head tilt, shoulder elevation, and spinal curvature—corresponded clearly and intuitively to the actual physical gestures. This practical cross-check confirmed that the system can reliably detect and represent the postural adjustments relevant to vocal pedagogy.

Following this pilot testing and a thorough examination of the collected data, the biomechanical relevance of the skeletal tracking system was confirmed, and its use was validated for application in the experimental setup (see Section 4.2).

## 4. Analysis and Results

The above approach describes the first (to the extent of our knowledge) skeletal tracking system, specifically designed for gesture-following in operatic singing, which can be applied to monitor and record a total of seven distinct body movements simultaneously.

The analysis of the experimental data was based on observations within the recorded data of the aforementioned 28 singers, while the examples presented within this paper have been selected in order to include participants representing all three experiment venues and all five experiment recording sessions (distinct days). Beyond that, for skeletal tracking verification purposes, the examples shown here depict participants selected from the singers group in such a way as to achieve inclusion of diverse heights/builds, as well as diverse attire/venue-wall color contrast degrees.

### 4.1. Face Validity Assessment of Captured Biomechanical Functions

**Figure 2:**
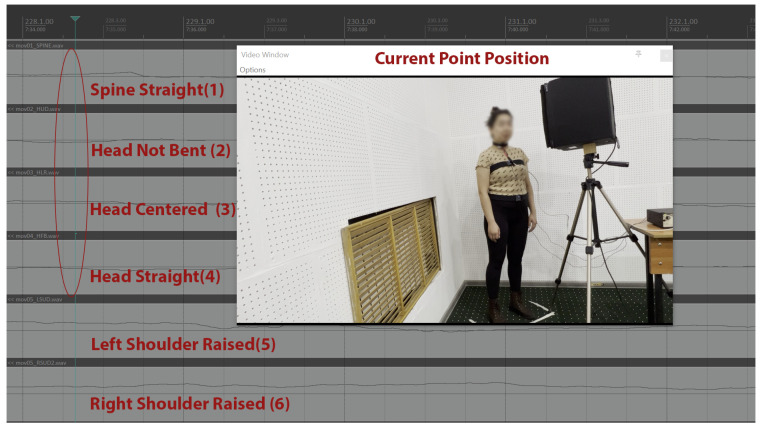
Kinect sensor data for the beginning of an Aria phrase of a singer. Marks on the figure represent: spinal (1), cervical (2)–(3)–(4), and shoulder (5)–(6) movements.

The sensor data streams from the singers were examined by author E.A., a vocal pedagogy expert with 22 years of classical singing teaching experience and a doctoral candidate (at the time) in vocal pedagogy research with comprehensive knowledge regarding the biomechanics of operatic singing. An example that illustrates the relevance of the captured data can be found in the excerpt analyzed in Figure 2.

For the Figures included within this paper’s analysis, Kinect data-stream zero values represent each participant’s self-adopted ‘natural upright’ specific joint relative position, defined during calibration, as per the posture the participant assumed when asked to adopt their individual, self-perceived, upright standing body alignment.

In Figure 2, the singer is singing an Aria and seems to have slightly raised shoulders (5)–(6) in comparison to their personal reference posture, a centered spinal posture (1), and head (2)–(3)–(4) position. The observed body pose reflects expected biomechanical parameters for operatic singing in a studio recording context, including mild postural engagement, vertical alignment, and controlled head positioning. All positional cues were also verified through the accompanying video recording.

**Figure 3:**
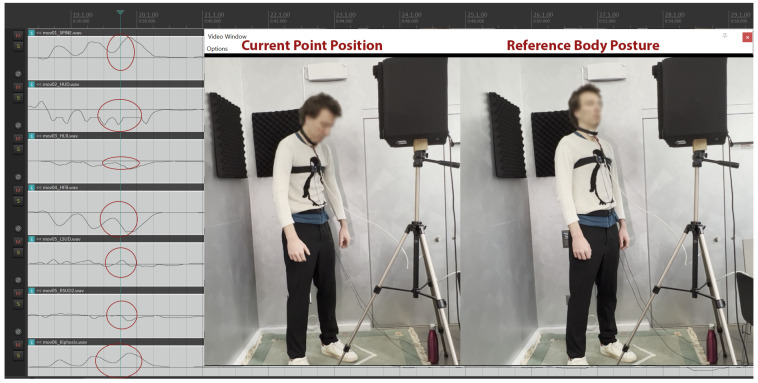
Skeletal tracking data streams for a singer performing the pre-trials sync events.

**Figure 4:**
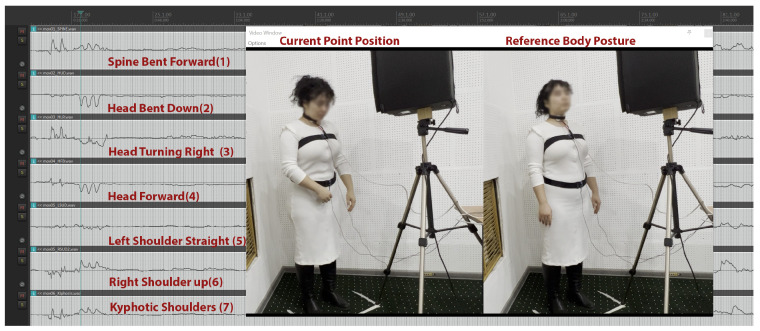
Skeletal tracking data streams for a singer performing the pre-trials sync events.

In addition to instances within conventional studio recording settings for classical singers, where normal positions were expected to prevail, moments of specific actions were also analyzed, such as the protocol synchronization events, (including a sharp cough performed with a head/body bend; see Figure 3 and Figure 4), during which the skeletal tracking system was expected to register relevant movement in the spine, shoulders, and head.

In Figure 3, one of the singers is shown during the pre-trials sync events, for the purpose of which singers were asked to perform three consecutive very soft “coughs” with closed lips, almost like a ‘throat clearing,’ with a simultaneous forward head bend. The synchronization of Kinect Azure sensor streams is visible at the zoomed-in three spikes in all relevant streams. The upper body position, in relation to the reference posture, is clearly described within the data streams, listed here as illustrated from top to bottom: (1) Spine bent forward, (2) Head bent downwards, (3) Head turning to the right, (4) Head advancing forward, (5) Left shoulder up, (6) Right shoulder straight, (7) Shoulders in a kyphotic forward shrug position.

Similarly, during their respective synchronization events (as described above), the singer depicted in Figure 4 seems to exhibit a body position that includes (1) spine bent forward, (2) head bent downwards, (3) head turning to the right, (4) head advancing forward, (5) left shoulder up, (6) right shoulder straight, and (7) shoulders in a kyphotic forward shrug position.

**Figure 5:**
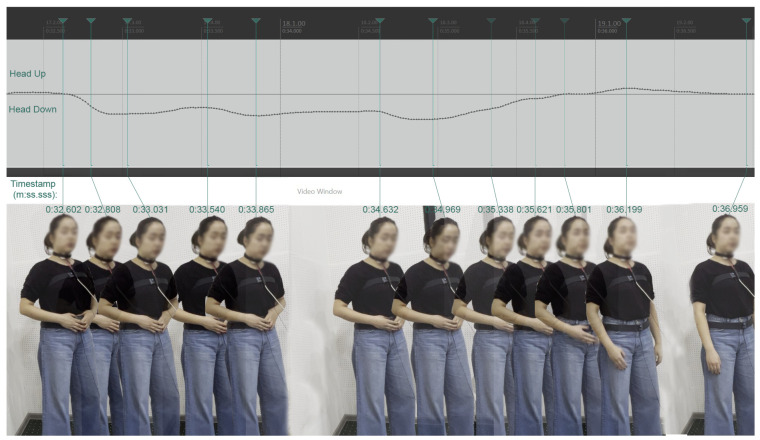
Time-ordered video frames of a single singer, corresponding to points on the graph of normalized head up/down relative position values. Zero value represents the participant’s self-adopted ‘natural upright’ head position.

**Figure 6:**
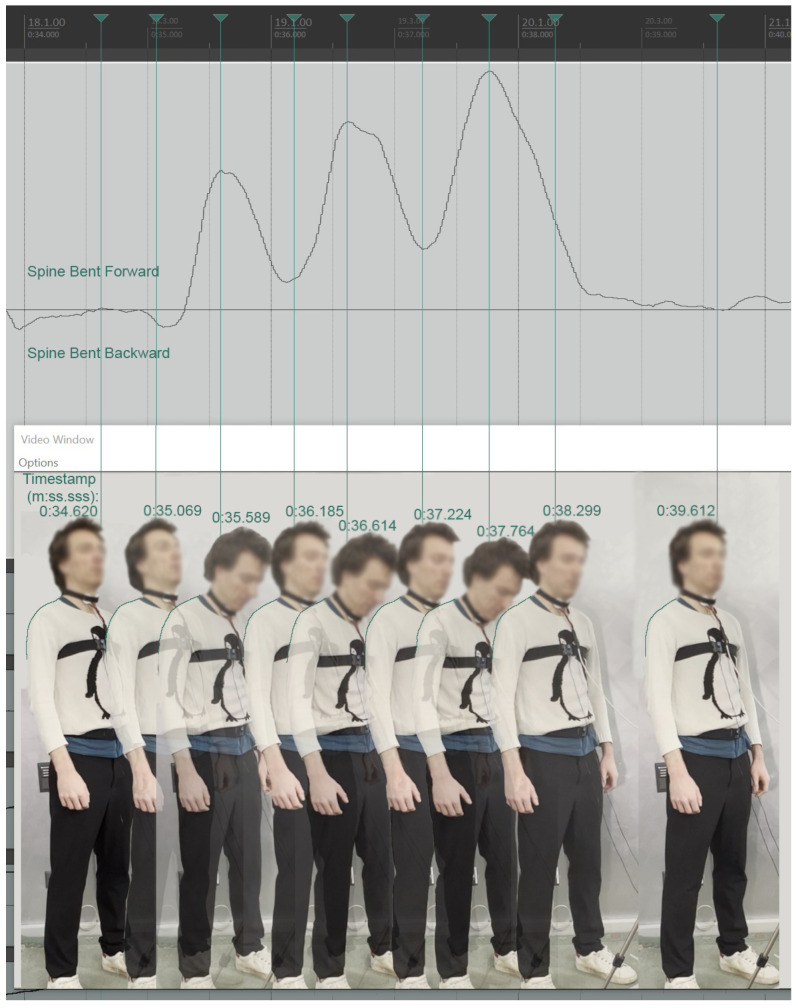
Time-ordered video frames of a single singer, corresponding to points on the graph of normalized spine forward/backward relative position values. Zero value represents the participant’s self-adopted ‘natural upright’ spinal position.

**Figure 7:**
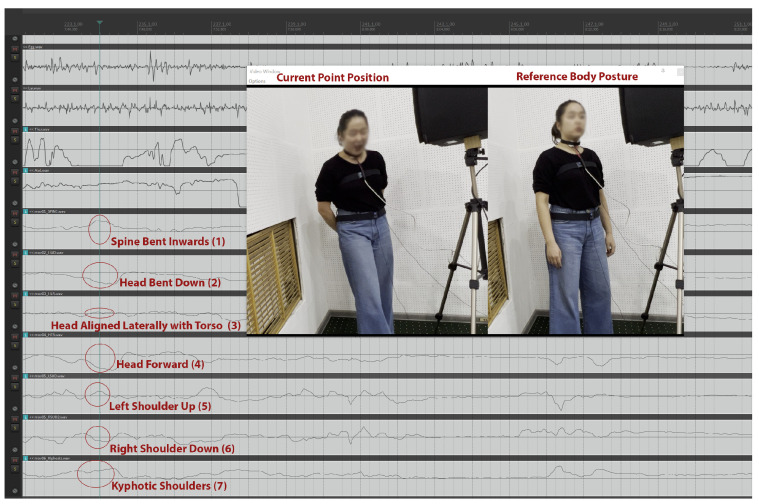
Skeletal tracking data streams for a singer at a position far from their reference body posture (during sensor calibration).

**Figure 8:**
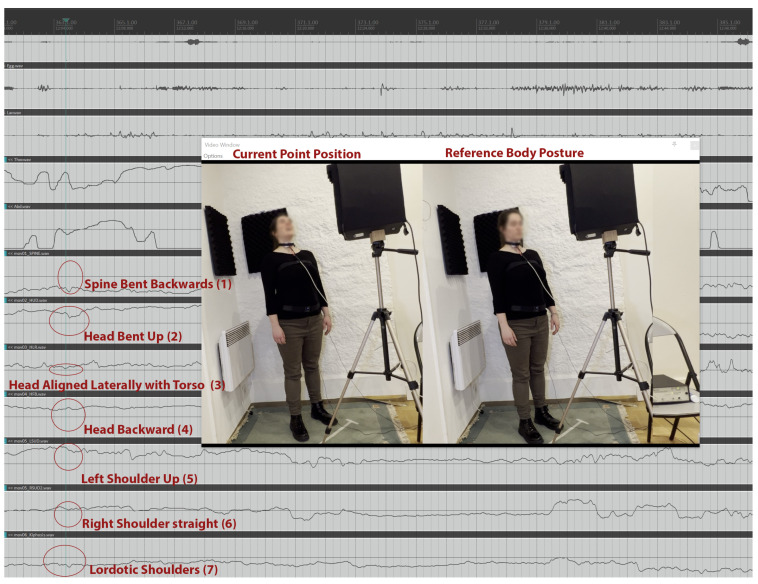
Skeletal tracking data streams for a singer at a position relatively far from their reference body posture (during sensor calibration).

The process described here accounts for how data streams from this sensor were examined in order to confirm that they reflect their corresponding biomechanical events, ensuring that skeletal movements were captured consistently. Additionally, comparison of these data points across the sensors established that the system effectively captures a coherent and synchronized (albeit still partial) picture of the complex biomechanics involved in singing (see Figure 3, Figure 4, Figure 5, Figure 6, Figure 7 and Figure 8). The examples illustrated above serve as representative cases, demonstrating the consistency of captured data across the broader participant pool. It was assessed that the data support high face validity, making them intuitively aligned with the goals of the study.

### 4.2. Skeletal Tracking Validation Through Benchmarking Against Independent Parameters

After the experimental recording sessions, a comparative validation approach was employed to provide proof regarding the accuracy of the novel skeletal tracking component within the prototype. A motion capture (video) system was used as a ground truth reference for body movements. Although videos are not traditionally used in singing research, their precision in recording image makes them a reliable benchmark for verifying the accuracy of the skeletal tracking algorithm. Movements during the experiments were captured simultaneously using both video and the skeletal tracking sensor, allowing for comparison and identification of any deviations.

The data synchronization process detailed in Section 3.5 was followed by the manual inspection and comparison of the skeletal tracking data with video footage of the singers, focusing mostly on timestamps with rapid, simultaneous movements in multiple tracked body joints. This visual inspection validated that the tracked movements corresponded to the actual physical movements observed in the video. The alignment of these two layers of validation (ground truth data and manual inspection) provided robust evidence for the reliability of the skeletal tracking algorithms (see Figure 3, Figure 4, Figure 5, Figure 6, Figure 7 and Figure 8).

In Figure 5, twelve time-ordered video frames of a single singer over a 4.4 s interval are shown, demonstrating the motion capture module’s ability to accurately record relative head up/down movements with respect to the torso in operatic singing under studio recording conditions. These images correspond to points on the graph of normalized head up/down values. The frames are drawn from the participant’s initial sensor synchronization sequence, during which they were instructed to perform three consecutive small ‘cough-like’ head-bending movements. Although this participant executed only very subtle head bends, these were successfully captured and are clearly represented in the graph, in some cases even more distinctly than in the video frames.

In a similar manner, the singer in Figure 6 is shown through nine time-ordered video frames of a participant over a 5 s interval, demonstrating the motion capture module’s ability to accurately record relative spine forward/backward bending movements with respect to postural alignment in operatic singing under studio recording conditions. These images correspond to points on the graph of normalized spine forward/backward values. The frames here are drawn again from the same sensor synchronization sequence detailed above. Although this participant executed only very subtle forward and backward spine bends, these seem to have been successfully captured and to be clearly visible in the graph.

As an additional example, Figure 5 displays skeletal tracking data streams for a singer at a position far from their reference body posture as captured during the initial pre-singing-protocol sensor calibration sequence. The depicted upper body position, in relation to the reference posture, is clearly described within the data streams: (1) the spine is bent inwards, (2) the head is bent downwards, (3) the head is almost completely aligned laterally with the torso—perhaps very slightly turned to the right—, (4) the head is advancing forward, (5) the left shoulder is raised, (6) the right shoulder is lowered, and (7) the shoulders are in a kyphotic forward shrug position. This specific case was among the clearest examples in the full dataset of 28 participants, where a singer noticeably deviated from their self-adopted reference pose. These deviations from the participant’s self-adopted ‘natural upright’ reference position provide an illustrative example of the system’s ability to detect and represent clear biomechanical differences in posture using the skeletal tracking data streams. This appears to be valid even in such cases when a singer assumes an unexpected or non-neutral posture, as was the case in this example, which had to be located by specifically searching within the calibration timeframes, since no such outlier poses were observed during the actual singing protocol.

Another example from the calibration sequence is shown below in Figure 8, which displays skeletal tracking data streams for a singer at a position relatively far from their reference body posture as captured during the initial pre-singing-protocol sensor calibration phase. The depicted upper body position, in relation to the reference posture, is clearly reflected in the skeletal tracking data: (1) the spine is bent backwards, (2) the head is bent upward (chin up), (3) the head is almost completely aligned laterally with the torso—very slightly turned to the right—, (4) the head is pulled backward, (5) the left shoulder is elevated, (6) the right shoulder remains in a neutral vertical position, and (7) the shoulders are positioned in a lordotic, backward-aligned posture. This case, like the previous one, further supports the ability of the skeletal tracking module to reliably capture and represent subtle yet distinct postural deviations from the participant’s reference alignment during the calibration period.

## 5. Discussion

The present study introduces an innovative skeletal tracking module for operatic singing research, integrated within a broader prototype that also includes audio, glottal, and respiratory sensors. Building on a growing demand for evidence-based vocal pedagogy, this approach offers a unique capability to objectively capture and synchronize relevant biomechanical parameters focused on posture and motion tracking. This discussion reflects on the pedagogical implications of the module’s technical achievements, evaluates its reliability and usability, and considers its potential as a foundation for future developments in science-informed operatic training.

The module represents an innovative approach to recording movement patterns relevant to operatic singing. To the best of our knowledge, it is the first such system integrated into an operatic singing research context to enable detailed tracking of postural and skeletal kinetic functions (including cervical, shoulder, and spinal movements) synchronized with acoustic and glottal data. The qualitative review of the module’s output confirmed the high face validity of our experimental measurements, indicating that the captured postural patterns corresponded with established pedagogical expectations. Likewise, concurrent changes captured across different movements during synchronized events (such as a deliberate cough paired with a head movement) illustrated a high degree of consistency, confirming that the multimodal system paints a unified picture of the singer’s physiology. The skeletal tracking data were also benchmarked against simultaneous video recordings (used as an external ground truth); this comparison showed that the Kinect-derived measurements faithfully matched the observed movements on video, lending strong evidence for the accuracy and reliability of the system.

Regarding future-proofing, the system is currently recording more biomechanical data than those utilized in the present evaluation experiments. More specifically, the collected orientation data are not being analyzed in the current project but remain available for future meta-analysis and/or the development of more robust gesture-recognition algorithms. There is also the possibility for the Kinect to record further gestural information beyond what is currently analyzed. This extensibility ensures that the platform evolves with emerging pedagogical questions or incorporates additional bio-signals, maintaining the long-term relevance of the system as a research and training tool.

The limitations of this module lie mainly within the small number of crashes in the Max/MSP software, leading to partial data loss from skeletal tracking for 3 (out of the total 28) participants. While this data loss was minimal, updates to this software component are already underway to address the issue. Additionally, the microphone and recording equipment of the prototype tool used in these recordings occluded a complete frontal view of the singer, necessitating a frontal-lateral placement at approximately 45°. Although previous studies have indicated that frontal placement yields higher accuracy in shoulder angle estimation for the shoulder occluded by the torso in lateral camera placement setups [17], our tests did not reveal qualitatively visible accuracy issues with the chosen 45° placement. Nevertheless, it is possible that a potential minor accuracy reduction could occur in cases of excessively rapid movements, conditions which are typically not expected within the scope of controlled experimental operatic recordings. Future refinements could focus on improving some of the above factors.

The continuous application of science and technology in operatic pedagogy reflects a broader educational trend towards interdisciplinary innovation. By proposing to ground voice pedagogical practices on high-quality scientific evidence, this research aims to ensure that operatic singing remains a dynamic and adaptive art form. The positive informal feedback reported by the experiment participants regarding the non-invasiveness of the employed skeletal tracking system emphasizes the transformative potential of the innovations presented here. Future work on this module may also explore utilization in different fields, such as designing pedagogical versions to support real-time analysis and vocal/postural monitoring.

Notably, this work responds to recent calls in the vocal pedagogy literature for more evidence-based training methods [4] and for pedagogical approaches that explicitly target physiological subsystems of the voice [2]. By quantitatively capturing elements like posture and head movement, the system provides a concrete link between traditional teaching and the underlying biomechanics of singing, allowing instructors to address technique adjustments with objective feedback rather than solely subjective observation. In practice, this could translate to more personalized and data-informed training interventions—for instance, giving a singer real-time visual feedback on their alignment or movement patterns—which may accelerate skill acquisition and promote healthier vocal habits [21].

## 6. Conclusions

This novel skeletal tracking method for singers could play a central part in innovating operatic voice research. It is designed to record joint data and estimate a wide array of relevant biomechanical parameters—spinal/postural, cervical, and shoulder movements—while also synchronizing these data with other acoustic and physiological signals, thus enabling an unprecedented scope of analysis for singing mechanics. The successful validation of this sensor within an operatic singing framework and our module approach demonstrates not only the technical feasibility of such integration but also its value in yielding objective insights into the nuanced physical behaviors of operatic singers. This motion capture addition lays a foundation for truly evidence-based vocal pedagogy, bridging the longstanding divide between the art of singing and the science of voice. By embracing advanced biomechanical feedback tools like this, future training programs can quantitatively inform pedagogical techniques, ultimately enriching the learning process and helping to preserve singers’ vocal health as they strive for artistic excellence.

## Data Availability

Data are available upon request.
